# Middleware for Plug and Play Integration of Heterogeneous Sensor Resources into the Sensor Web

**DOI:** 10.3390/s17122923

**Published:** 2017-12-15

**Authors:** Enoc Martínez, Daniel M. Toma, Simon Jirka, Joaquín Del Río

**Affiliations:** 1 SARTI research group, Electronics Department, Universitat Politècnica de Catalunya, 08800 Vilanova i la Geltrú, Spain; daniel.mihai.toma@upc.edu (D.M.T.); joaquin.del.rio@upc.edu (J.D.R.); 2 52° North Initiative for Geospatial Open Source Software, 48151 Münster, Germany; jirka@52north.org

**Keywords:** Sensor Web Enablement, plug and play, interoperability, SensorML, Open Geospatial Consortium, sensor integration, OGC PUCK protocol

## Abstract

The study of global phenomena requires the combination of a considerable amount of data coming from different sources, acquired by different observation platforms and managed by institutions working in different scientific fields. Merging this data to provide extensive and complete data sets to monitor the long-term, global changes of our oceans is a major challenge. The data acquisition and data archival procedures usually vary significantly depending on the acquisition platform. This lack of standardization ultimately leads to information silos, preventing the data to be effectively shared across different scientific communities. In the past years, important steps have been taken in order to improve both standardization and interoperability, such as the Open Geospatial Consortium’s Sensor Web Enablement (SWE) framework. Within this framework, standardized models and interfaces to archive, access and visualize the data from heterogeneous sensor resources have been proposed. However, due to the wide variety of software and hardware architectures presented by marine sensors and marine observation platforms, there is still a lack of uniform procedures to integrate sensors into existing SWE-based data infrastructures. In this work, a framework aimed to enable sensor plug and play integration into existing SWE-based data infrastructures is presented. First, an analysis of the operations required to automatically identify, configure and operate a sensor are analysed. Then, the metadata required for these operations is structured in a standard way. Afterwards, a modular, plug and play, SWE-based acquisition chain is proposed. Finally different use cases for this framework are presented.

## 1. Introduction

As the interest of studying global environmental phenomena is growing, collaborative research environments are becoming more important. In these environments, data coming from many different sources has to be combined and managed in a standard way in order to avoid information silos. The lack of standardization and data harmonization across scientific domains and scientific data infrastructures has been the driving force for the Open Geospatial Consortium (OGC) to propose the Sensor Web Enablement framework (SWE) [1]. This framework is a suite of data model, encoding, and interface standards which aim to provide the building blocks for interoperable Sensor Web infrastructures. In this context, the concept of the Sensor Web refers to a set of Web accessible sensor networks and their collected sensor data/metadata that can be discovered and accessed using standard protocols and application programming interfaces [2].

**Figure 1:**
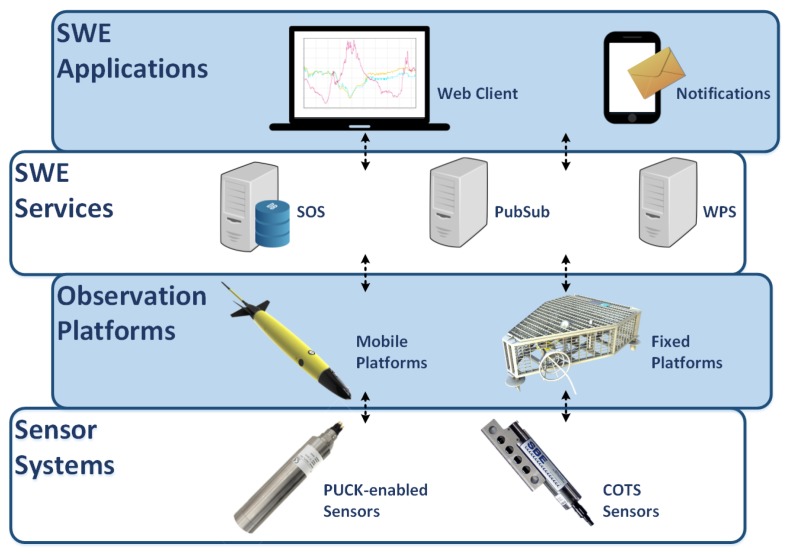
Sensor Web Enablement layer stack for ocean observing systems. The sensor layer comprises the physical sensors. The integration layer includes all the mechanisms that bridge the data from the sensor’s output to Sensor Web services. The Sensor Web Services layer is the core of a Sensor Web infrastructure, where the data is archived, processed and analysed. Finally the application layer provides interfaces between the Sensor Web services and the final users (e.g., data viewers).

The different components in an SWE-based architecture can be classified according to their role in a Sensor Web layer stack [3]. In Figure 1 a Sensor Web layer stack for ocean observing systems is presented. Although the interaction patterns in the upper layers of the SWE stack are strictly specified by the SWE standards, integrating sensors into Sensor Web services is not fully defined: While the transactional operations of the OGC Sensor Observation Service already allow the standardized publication of data, certain aspects such as sensor discovery and plug and play mechanisms are not yet sufficiently available. Therefore, bridging between the physical sensors and the Sensor Web services is still a challenge [4]. Many SWE-based architectures currently in operation still rely on proprietary mechanisms to integrate sensor data streams (e.g., by customizing SWE services to use existing observation databases as data source).

In ocean observing systems, instruments and sensor systems (in short: sensors) are commonly deployed using observation platforms, which are in charge of operating sensors and acquiring their data. The ocean observing community uses a vast collection of observation platforms, such as fixed underwater observatories, buoys, underwater gliders, autonomous surface vehicles, profilers, etc. Some of them are powered by batteries and use satellite communication links (i.e., underwater gliders), while others may be connected to the electrical grid and use broadband Ethernet (underwater cabled observatories). Due to their heterogeneous nature, observation platforms present a wide variety of architectures, resulting in a wide range of non-standardized protocols, data encodings, and communication interfaces.

Due to this heterogeneity, in most cases a specific driver is needed for each sensor-platform combination in order to gather data. Sensor manufacturers usually provide drivers for desktop environments (i.e., Microsoft Windows). However, marine sensors are frequently integrated in embedded environments where the provided drivers cannot be used [5]. If a new sensor has to be integrated or an existing sensor has to be moved from one platform to another, new specific components have to be developed. Generating a specific driver for each sensor is a time-consuming task that requires in-depth knowledge of both sensor’s protocol and the observation platform’s architecture [5].

**Figure 2:**
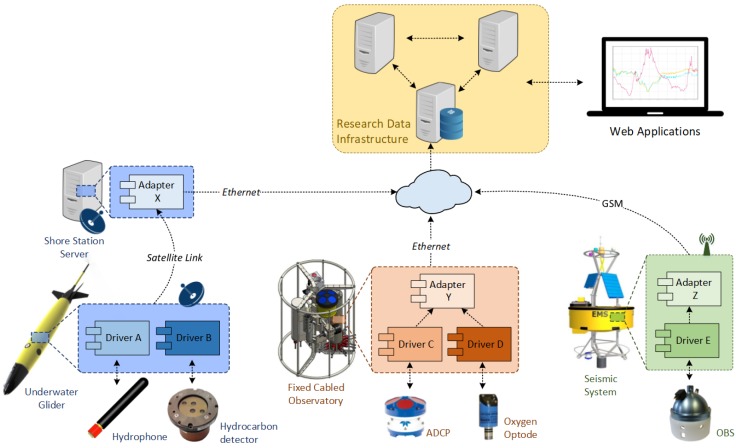
Collaborative research scenario using specific drivers and data converters for each sensor-platform combination, in this case an underwater glider, a buoy and a cabled underwater observatory.

Moreover, when integrating several platforms into SWE-based data infrastructures, each platform-specific encoding needs to be adapted by service adapters. This conversion can either be achieved directly on the platform or by an intermediate process on a land station server in the case of resource-constrained platforms. Figure 2 depicts a non-standardized scenario where different sensors are deployed using different observation platforms. As the number of platforms and sensors deployed within a collaborative environment grows, the number of custom components increases, as well as the infrastructure maintenance costs. Therefore, improving the interoperability among the infrastructure’s components should be a matter of great concern. Interoperability in this sense can be defined as the ability of two systems to exchange information and to interpret the information that has been exchanged [6].

The ultimate goal of interoperability in a collaborative research environment should be to enable the plug and play integration of sensor resources into observation platforms and data infrastructures, reducing the human intervention to a minimum. This goal implies two different levels of interoperability: operational and data management. From the operational interoperability point of view, a sensor should be automatically detected, configured and its data gathered by the observation platform’s acquisition process, as soon as it is deployed into an observation platform. From the data management side, the data retrieved from the sensor should be encoded in a standard format, compatible with the data infrastructure. Sensor metadata plays a key role in the acquisition. If managed properly it will enhance not only the interoperability of the sensor, but also its data traceability and ease further data quality procedures, allowing to pinpoint for example sensor malfunctions [7]. Therefore, metadata should be added alongside sensor data, providing contextual information [8].

**Figure 3:**
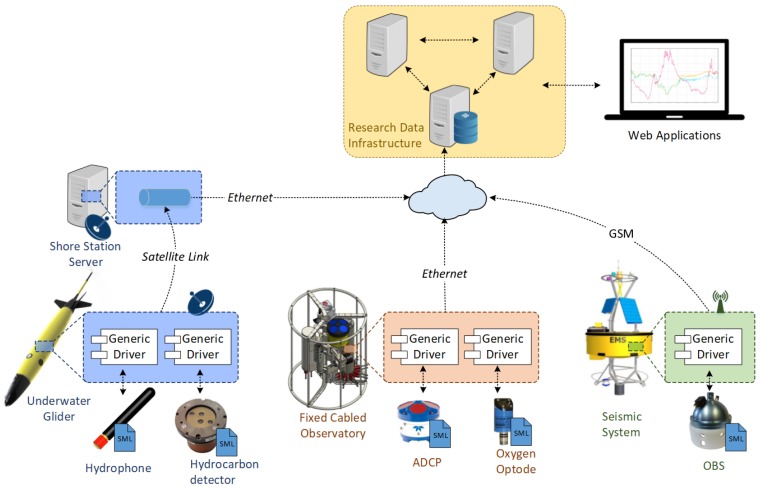
Proposed Sensor Web Enablement (SWE)-based architecture. Sensor resources are integrated by combining a standards-based description with a generic driver in order acquire data. As the generic driver provides a standardized output, the generated data can be directly sent to the SWE services using the observation platform’s communication link.

Within this work, a standardized, plug and play acquisition chain capable of bridging sensor resources to Sensor Web services is envisioned, implemented and evaluated. Instead of using a specific driver for each sensor-platform combination, the use of a generic driver component configurable through OGC standards is proposed. In order to abstract the peculiarities of each sensor, this approach makes use of a metadata file with a machine-understandable unambiguous sensor description, encoded in a standard way. Afterwards, the generic driver should be able to access such a sensor description file and configure itself accordingly, being able to operate a sensor without any a priori knowledge of the device nor the observation platform where it is hosted. The gathered sensor data should be encoded in standardized SWE formats, ensuring the plug and play integration of sensors into SWE services. This generic component should also have its own auto-description methods, in order to make their role understandable to both humans and machines. The envisioned architecture is presented in Figure 3.

### Related Work

The SWE framework aims at tackling a huge interoperability challenge for middleware approaches since heterogeneous devices are expected to collaborate together in communication and information exchange. Implementations of Sensor Web Enablement standards across different domains have found difficulties when integrating sensors into SWE services. Walter and Nash analysed the difficulties of integrating sensors and proposed the use of lightweight SWE connectors (custom drivers) in order to bridge from sensor-specific protocols to SWE services [9]. This approach has been widely used in different applications, using custom drivers or plug-ins as SWE connectors [10,11].

A slightly different approach is to enhance each sensor controller with embedded Web services in order to archive and exchange data in a standardized manner with the components in the upper layers of the Sensor Web Enablement stack. This sensor integration strategy has been used in different fields, such as precision agriculture applications [12] and air quality monitoring [13,14]. This approach, although it provides a good interoperable interface with other Sensor Web-enabled components, does not directly solve the challenge of integrating new sensors into a platform as it still relies on custom drivers.

Another issue that arises when embedding SWE services into sensors controllers, is that SWE services largely rely on exchanging eXstensible Markup Language (XML) files [15], which can be very verbose and require a significant amount of resources to process. When integrating Web services on resource-constrained devices in terms of computational power or communication bandwidth, the use of plain XML may not be suitable. Different strategies on the optimization on the information exchange have been studied in [16]. The Efficient Extensible Interchange format showed promising results in terms of required computational resources and information compactness [17].

To overcome the presented limitations and address these integration challenges, the IEEE 1451 standard proposed the idea of a smart sensor [18]. Smart sensors are defined by the IEEE 1451 standard as sensors with small memory and standardized physical connection to enable the communication with data network and processor. This standard has also been applied to bridge sensors to SWE services [19]. However, this approach assumes an IEEE 1451 compliant sensor device. A broad implementation of this standard has not yet been achieved (the vast majority of commercial sensors do not support IEEE 1451) so that most sensors cannot be integrated using this approach. Moreover, the IEEE 1451 compliant sensor devices are limited by the lack of flexibility, absence of customization options, narrow spectrum of applications, and the basic communication protocol.

A more recent approach is the OGC SensorThings, dedicated especially to Internet of Things (IoT) sensors [20], where sensors are envisioned as web-accessible devices. However, in real world scenarios sensors may be deployed in remote and inaccessible observation platforms where internet connectivity cannot be guaranteed.

As in real world applications a huge variety of sensor protocols (standardized or proprietary) are utilized, another approach is to address the interoperability gap from the opposite direction, by introducing mechanisms to abstract from the variety of sensor protocols, such as Sensor Abstraction Layer (SAL) [21] or Sensor Interface Descriptor (SID) [22].

The Sensor Abstraction Layer (SAL), which describes all the sensors in a uniform manner, makes use of and expands the SensorML 1.0 standard to describe sensor interfaces and commands. It hides the specific technological details by matching sensor-specific commands to generic SAL commands. However, it still relies on a plug-in approach to integrate new sensors [23].

Similar to SAL, the Sensor Interface Descriptor (SID) model extends SensorML 1.0 to formally describe a sensor’s protocol. The generated sensor interface description is used as a platform independent sensor driver which contains the necessary information to integrate a sensor on demand by translating between sensor protocol and Sensor Web protocols. A benefit of SID is the availability of open-source components to generate SID descriptions [24] as well as a SID interpreter middleware based on Java. Several applications of SID can be found at the literature [5,25,26].

However, a still remaining open challenge is to practically include such a universal approach in the Sensor Web middleware of marine observatory platforms. A Java-based middleware is a good approach to abstract on the operating system level, however, it may not suitable to be deployed in resource-constrained platforms. Furthermore, the SID model extension defines the whole Open Systems Interconnection (OSI) model stack for each sensor command, resulting in very verbose XML files, which are rather difficult to interpret.

In this work the focus is put on semantic interoperability, emphasizing the need of standard middleware components compatible with different observation platforms. The rest of this paper is structured as follows: Section 2 discusses the requirements to achieve a true plug and play sensor integration into existing data infrastructures. Section 3 presents an SWE-based universal acquisition chain. Section 4 presents Sensor Deployment Files (SDF) as a standard way to organize all the metadata related to a sensor deployment and how it can enhance interoperability. In Section 5 the SWE Bridge middleware is presented, a universal plug and play sensor data acquisition middleware. Finally in Section 6 different use cases of the acquisition chain are presented.

## 2. Sensor Integration into Observation Platforms

### 2.1. Requirement Analysis

When integrating new sensor resources into existing data infrastructures, several standardized operations are required. These operations can be classified in two different levels: Instrument Level, and Sensor Web level [5]. The Instrument Level operations are related to the sensor’s operational challenges, focused on the sensor-platform interaction, including both sensor configuration and sensor data retrieval. The Sensor Web challenges are related to the sensor data management, including standardized data encodings, sensor data discovery, tasking mechanisms, etc.

According to previous work on this topic, at least four operations are required at the instrument level: sensor detection, sensor identification, sensor configuration and simple measurements operations [5]. These are operational requirements, focused on the direct sensor-platform interaction.

From the data management side, there is a multitude of operations that can be realized, such as data discovery, data access, sensor tasking, events and notifications, etc. However, this work focuses on the integration of sensors to the Sensor Web from a platform operator point of view. Thus only the sensor registration and sensor data ingestion operations are considered within this work.

Some ocean observation platforms present additional constrains such as limited power availability and low-bandwidth communications. These platforms are usually based on low-power embedded systems, with limited computational capacity. Thus, a sensor plug and play mechanism should take into account these constrains. Summarizing, the requirements to achieve plug and play integration into observation platforms are:

1. **Sensor Detection**: Detect a new sensor when it is attached to an observation platform. The host platform controller should be able to detect a new sensor without human intervention.
2. **Sensor Identification**: Obtain an unambiguous description of the sensor, including all the metadata to identify the sensor (unique ID, sensor model, etc.) and all information required to register the sensor to an existing Sensor Web server.
3. **Sensor Configuration**: This requirement addresses all operations required before the platform can start retrieving data from a sensor. This includes establishing a communication link between the platform and the sensor and applying any configuration required by the sensor (i.e., activate a specific acquisition channel, set the sampling rate, etc.).
4. **Simple Measurements Operations**: Those operations that are directly related to the retrieval of data. These operations may be actively querying the sensor for data or listening to data streams. Also knowledge of the data interface provided by the sensor is required in order to parse, process and store the data.
5. **Sensor Registration**: Registering a sensor to existing Sensor Web server requires a considerable amount of metadata organized and structured in a coherent way, including physical parameters that are being measured (observable properties), computational representation of the real-world feature that is being measured (feature of interest), alongside with other sensor characteristics. Furthermore, the meaning of this metadata has to be made explicit and understandable by machines, thus, controlled vocabularies containing formal definitions shall be used [27].
6. **Data Ingestion**: Once the sensor is registered, the data measured by this sensor has to be ingested to the server, where it will be archived.
7. **Resource Constrains**: Any plug and play mechanism aimed to integrate sensors into marine data acquisition platforms should be able to work in low-bandwidth, low-power and computationally-constrained scenarios.

### 2.2. Protocols and Standards

**Figure 4:**
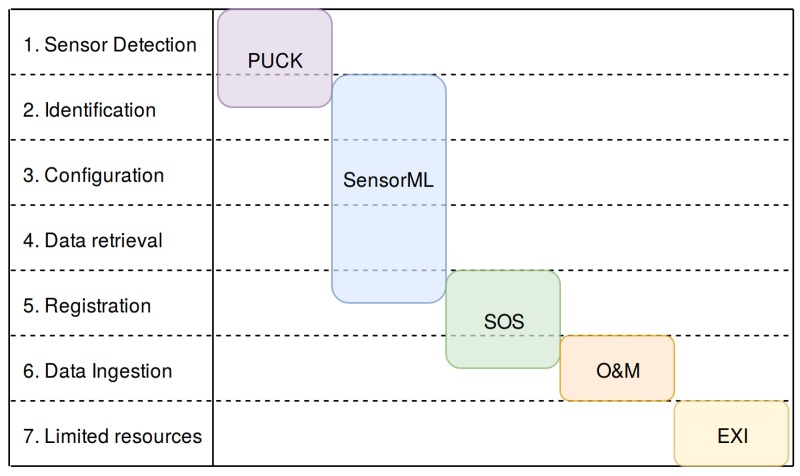
Requirements for a plug and play mechanism (rows) and protocols/standards that can fulfil these requirements (columns).

In order to fulfil the previously presented requirements in a standardized way, a set of protocols and standards suitable of fulfilling these needs are presented in this section. The SWE framework provides a set of standards and protocols that can fulfil these needs, as shown in Figure 4.

#### 2.2.1. OGC PUCK Protocol

The sensor detection requirement can be fulfilled using the OGC PUCK protocol within the acquisition platform controller [28]. This protocol is an add-on that can be implemented in any serial or Ethernet sensor alongside with any proprietary protocol, rather than replacing it. This protocol defines a set of commands that grant transparent access to an internal memory, named *OGC PUCK payload*. This payload is frequently used to store sensor metadata. Another key feature of the OGC Puck protocol is its *softbreak* operation, which provides on-the-fly detection without any prior knowledge of the sensor, fulfilling requirement 1.

#### 2.2.2. Sensor Model Language

The OGC Sensor Model Language (SensorML) permits to encode detailed sensor descriptions within an XML file [29]. Its main goal is to enhance interoperability, making sensor descriptions understandable by machines and shareable between intelligent nodes. Moreover, additional information related to specific deployments can also be encoded using this standard. Thus, both sensor configuration and measurement operations can be addressed with the SensorML standard.

It is highly flexible and modular as it can describe almost every sensor related property or sensor-related process. However this flexibility and modularity can prove a double-edged sword, as the same information can be encoded in different ways, increasing the difficulty to generate smart processes capable of interpreting SensorML definitions. For this reason there is ongoing work to develop marine SWE profiles of SensorML that define more precisely how this standard should be applied in ocean observing applications [30].

By combining the OGC PUCK protocol and a SensorML description file, it is possible to automatically detect a sensor and retrieve its description encoded in a single standardized file without any a-priori knowledge about the sensor. If an interpreter software can interpret this file, it can identify the sensor, configure it and retrieve its data, meeting the requirements 2, 3 and 4.

#### 2.2.3. Sensor Observation Service

The SOS standard provides the set of operations required to provide access sensor observation data/metadata as well as to register and archive sensor data and metadata within a data repository [31]. Due to its role as a data and metadata archive, this standard is a key piece of any Sensor Web infrastructure, providing support for requirement 5, sensor registration.

#### 2.2.4. Observations and Measurements

The Observations and Measurements (O&M) standard specifies an abstract model as well as XML encoding for observations and related data, such as features involved in the sampling process [32]. It provides an uniform and unambiguous way to encode sensor measurements, fulfilling the requirement 6.

#### 2.2.5. Efficient XML Interchange

The Efficient XML Interchange (EXI) is a World Wide Web Consortium’s (W3C) format that enables the compression of large ASCII XML files into efficient binary files, significantly reducing its size. This format has been designed to allow information sharing between devices with constrained resources, meeting requirement 7 [17].

## 3. SWE-Based Acquisition Chain

**Figure 5:**
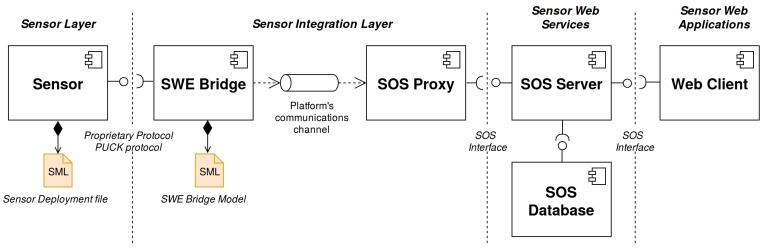
Proposed SWE-based acquisition chain.

Using the standards and protocols presented in the previous section, an SWE-based, plug and play acquisition chain deployable in a wide variety of ocean observing systems is envisioned. A set of interoperable standard components are proposed in order to bring data from the sensor itself to the Sensor Web, regardless of the constraints of the platform where the sensors are deployed. Figure 5 shows the proposed acquisition chain with its components, classified within the Sensor Web Layer stack. In order to make this architecture suitable for a wide range of scenarios, emphasis has been put on open source software components as well as cross-platform implementations. Each sensor has its own associated Sensor Deployment File (SDF), which encapsulates an unambiguous description of the sensor. The SWE Bridge interfaces the sensor, discovering, operating the sensor and storing its data in standard O&M data files. It also has a SensorML description file, where all its functionalities are described. The files generated as SWE Bridge’s output are passed to the SOS Proxy using the observation platform’s communication channel (dependent on the acquisition platform). Finally the SOS Proxy injects the O&M data to the SOS server by using its SOS interface. The SOS server archives this data into a SOS database and also provides an interface to access the archived data in a standard manner for further processes, i.e., data visualization web clients.

### 3.1. Sensor

Sensors are the component gathering data and making it available through a communication interface, usually serial port or Ethernet. Three different kinds of sensors are taken into account within this work:

- **COTS Sensors**: Commercial off-the-shelf (COTS) sensors are commercially available devices without any particular enhancement in terms of interoperability.
- **OGC PUCK-enabled Sensors**: Sensors which implement the OGC PUCK protocol.
- **Virtual Instruments**: Software components that merge or process data from different sources, generating new data sets accessible through a communication interface.

Each sensor should have its own associated SDF, a SensorML description containing both sensor metadata and deployment information (see Section 4) regardless of their nature. This file is a key piece of the architecture, as it contains all metadata required to describe, operate and register a sensor into Sensor Web services. OGC PUCK-enabled sensors may have their own SDF embedded within its internal payload memory, providing automatic detection and self-description capabilities.

Ideally physical sensors should be OGC PUCK-enabled to provide end to end plug and play capabilities. However, the majority of commercial sensors do not implement this protocol and do not provide auto-detection and self-description procedures. Therefore the operators have to manually match the sensor with SDF stored locally on the platform in order to provide compatibility with the proposed standardized architecture.

### 3.2. SWE Bridge

The SWE Bridge is a universal acquisition middleware, aimed to be deployed in any acquisition platform, regardless of its software and hardware architecture. It interprets SDFs, automatically configuring itself to connect to and operate a sensor. Its implementation of the OGC PUCK protocol allows to automatically retrieve a SDF from the sensor itself, enhancing interoperability and providing a plug and play mechanism. When interfacing with a COTS a local SDF has to be used. The acquired data is stored in standard O&M files, encoded in XML or EXI format in order to reduce their size [17].

The observation platform’s communication link to a shore station can be critical in terms of bandwidth as well as in terms of power consumption. Therefore the SWE Bridge does not directly send the O&M files to the server, but relies on the platform’s operator to setup for this transmission. The platform operator can decide under which conditions is desirable to transmit these files (i.e., stopping the transmission when the battery is low). Thus the platform operator still has full control of the platform.

The SWE Bridge has its own SensorML description, the SWE Bridge Model, where all its functionalities, parameters and built-in functions are described in a standard manner. This model allows an automated process to understand the role and the capabilities of the SWE Bridge within the acquisition chain.

### 3.3. SOS Proxy

The main functionality of the SOS Proxy is to decouple the SOS transactions from constrained platforms without Internet gateway, such as satellite-based platforms. It acts as a transparent intermediary layer, which takes O&M files from the platform’s communications channel (i.e., satellite link) and forwards them to an SOS Server. As the SOS Proxy is completely transparent to the rest of the architecture, it does not have an associated SensorML description.

Depending on the nature of the observation platform, the SOS Proxy can be deployed in the platform itself (i.e., a platform with direct Internet access through a GSM modem), otherwise it can be deployed in a shore station server (i.e., platform with proprietary satellite communications). Open source implementations of this software component are available in Java and Bash [33,34].

### 3.4. SOS Server

The Sensor Observation Service (SOS) acts as a server for storing and managing both sensor data and metadata. Due to its data archiving and metadata management roles, as well as its ability to interface with further services, it is one of the core components of the proposed SWE-based cyber-infrastructure, vital to interact with further processes.

In the proposed acquisition chain the 52° North’s open source SOS implementation running on a PostgreSQL database is used [35]. For ingesting metadata about a sensor into an SOS server (registering a new sensor) the transactional *InsertSensor* operation of the SOS interface is used. The upload of data to the SOS server is achieved through the so called *ResultHandling* operations of the SOS standard (*InsertResultTemplate* and *InsertResult*). To ensure an efficient data transfer the server supports the insertion of EXI-encoded O&M files.

The SOS Server also provides a standardized interface for further process to query and access sensor data archived in the SOS database. SWE services can connect to the SOS server to query for archived sensor data and related metadata.

### 3.5. Web Client

In order to demonstrate the end-to-end integration a Sensor Web visualization client was developed. For this purpose the 52°North Helgoland Sensor Web viewer is used, which is an open-source, lightweight Web application that enables the exploration, analysis and visualization of sensor web data [36]. To support marine application, Helgoland is designed to support different types of platforms (i.e., stationary and mobile) as well as different types of observation data (e.g., time series, profiles, trajectories).

## 4. Sensor Deployment Files

The OGC SensorML standard can provide a robust and semantically-tied description of a sensor, including its metadata, communication’s interface and command set. However, a sensor can be a complex system configurable in different ways, depending on the deployment and its desired behaviour (i.e., change the sampling rate, select a specific acquisition channel, etc.). All these operations are not only related to the sensor description, but also to the desired acquisition process itself. Thus, alongside the sensor’s description there should be a description of the desired acquisition process for each deployment.

In order to enable on-the-fly integration of complex sensor systems into observation platforms, the Sensor Deployment Files (SDF) are introduced. These files, based on the SensorML standard, compile and organize in a coherent manner the sensor’s metadata and an accurate description of the desired acquisition process for a specific sensor deployment. They allow an interpreter software, such as the SWE Bridge, to automatically configure the sensor, retrieve its data and store it in standard O&M files. A SDF should contain at least the following information in order to enable plug and play capabilities:

- **Identification**: Provide the required identifiers for the sensor, such as unique ID, model, name, manufacturer, etc. Define which physical parameters is the sensor able to measure.
- **Communications Interface**: An unambiguous and accurate description of the sensor’s communication interface to allow an interpreter software to automatically establish a communication link without any a priory information about of the sensor.
- **Communication protocol**: Set of commands required to operate the instrument. This includes configuration commands and measuring operations, as well as a description of the encoding of the sensor outputs.
- **Operation**: Detailed description of the sensor operation, including which operations need to be executed, in which order, which post-processing procedures will be applied to the sensor data and how this data will be stored.

Alongside with this operational information, data management metadata can be included (where the sensor is deployed, in which platform is deployed, etc.). This metadata would allow further processes to interpret a SDF and automatically register the described sensor to a SOS instance without human intervention. This metadata is modelled as optional information in the Sensor Instance and the Sensor Model diagrams. Additional information such as calibration, deployment history or event contact list could also be added to a SDF.

**Figure 6:**
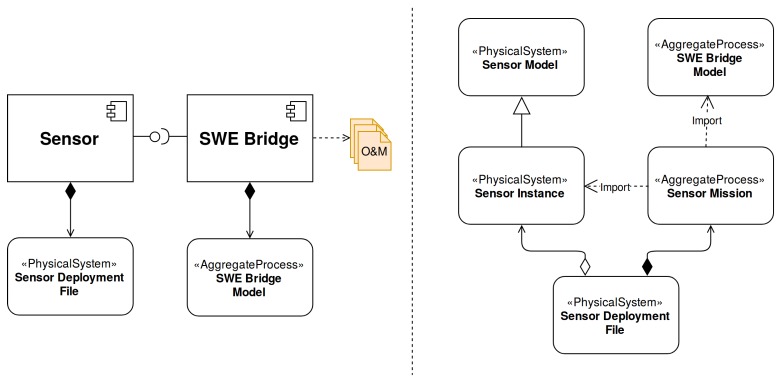
Sensor and SWE Bridge with their models (**left**) and Sensor Deployment Files (SDF) model (**right**). The Sensor, with its associated SDF, is interfaced by the SWE Bridge middleware, which also has its own Sensor Model Language (SensorML)-based description, the SWE Bridge Model.

A SDF is composed by a Sensor Instance (sensor’s metadata, inherited from a Sensor Model) and a Sensor Mission, as shown in Figure 6. The Sensor Mission uses the descriptions of both the Sensor Instance and the SWE Bridge Model (see Section 5.2) in order to arrange the available functionalities, defining how the acquisition chain should be configured.

The potential of SDF is its ability to describe a sensor and configure an acquisition chain with a single, standards-based file. Furthermore, as all the components related to the acquisition chain are described in a formal way, an automated system could process and understand these descriptions and automatically generate new SDF files to setup and update acquisition chains. A set of example SDF can be found at [37].

### 4.1. Sensor Model

**Figure 7:**
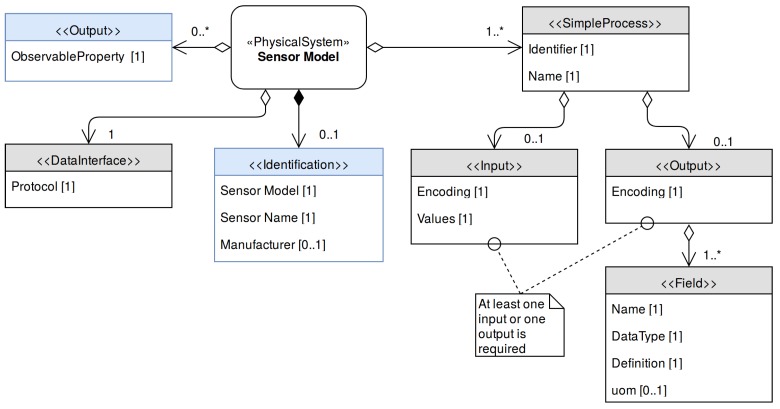
UML diagram of the Sensor Model. The gray elements represent all required information by the SWE Bridge (compulsory) while the blue elements represent optional metadata (used to register the sensor to a Sensor Observation Service (SOS) Instance).

The Sensor Model is a generic SensorML description of a family of sensors having common characteristics. As shown in Figure 7, it should include the sensor’s command set, its communications interface and other information applicable to all the sensors that share this model.

The *DataInterface* element is used to model the communication interface. As the interface parameters may depend on each deployment of the sensor, only the available communication protocols are defined at the Sensor Model stage.

A key aspect of a sensor model is the description of the sensor’s set of commands. Each command is described with a SensorML’s *SimpleProcess* element. At least one *SimpleProcess* is required in a Sensor Model (a sensor should at least provide one operation to retrieve data). The inputs of these processes define the command that the sensor expects, while the output corresponds to the sensor response to that command. If a sensor command does not have a reply to a specific command, the *SimpleProcess* will only have an input and no output. On the contrary, if the sensor streams data periodically the *SimpleProcess* used to model it will not have any input, but will have an output representing this stream. To model a command where the sensor responds to a specific command, both input and output need to be included.

Both input and output have an *encoding* section which describes how their contents (e.g., parameters, output values) are encoded. The output also contains an encoding element alongside with an array of fields, which are used to model the sensor response. Each field has a name, a *description* (reference to a controlled vocabulary) and a *type*, which corresponds to the SWE Common Data model encoding used to model this value [38]. When a physical magnitude is described, the units of measurement should be specified using the *uom* (units of measurement) element.

### 4.2. Sensor Instance

**Figure 8:**
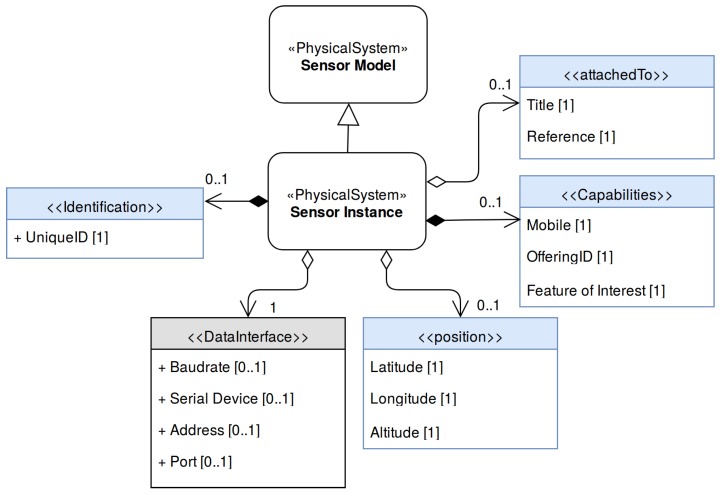
Sensor Instance UML diagram. It inherits a sensor model and expands it with information related to a sensor instance alongside with information related to a specific deployment. The gray elements represent all the required information by the SWE Bridge (compulsory ) while the blue elements represent optional metadata (used to register the sensor to a SOS Instance).

The Sensor Instance models a specific sensor by inheriting a Sensor Model and expanding it with static metadata related to a particular instance (such as unique ID) alongside with dynamic deployment information (such as position). A sensor and its host observation platform may be deployed in remote regions with low bandwidth communication, or no communication link at all. Therefore, a Sensor Instance cannot reference an on-line resource containing its Sensor Model description. Instead it expands a sensor model, resulting in a single file containing all the sensor’s metadata. Its model is shown in Figure 8.

From the operational point of view, the more important part of the Sensor Instance is the *DataInterface*. It expands the generic definition of the Sensor Model, defining the parameters required to establish a communication link from the observation platform to the sensor.

In the Sensor Instance model it is possible to include different elements that may be useful to register, discover and exploit the generated data (blue elements). Some of them are the *UniqueID*, *attachedTo* (reference to the observation platform where the sensor is deployed), position (spatial position where the sensor is deployed), *FeatureOfInterest* (a computational representation of the real world phenomenon being observed by the sensor).

### 4.3. Sensor Mission

**Figure 9:**
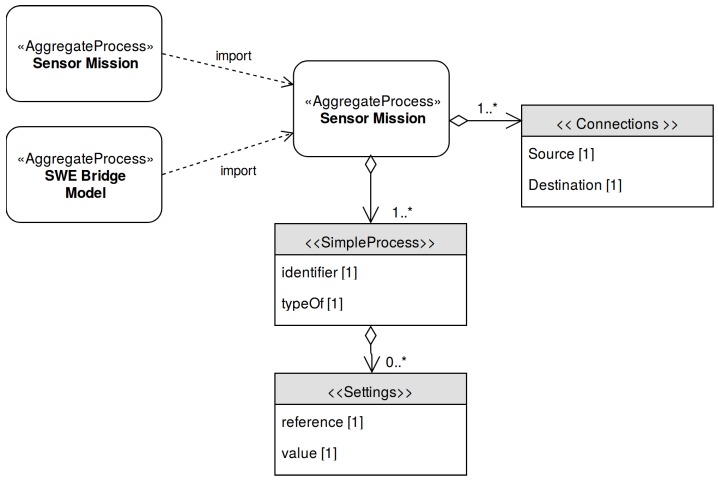
Sensor Mission UML Diagram.

The Sensor Mission models the desired acquisition process for a specific sensor deployment. This mission will be interpreted by the SWE Bridge or another implementation of a SDF interpreter, which will setup the acquisition process accordingly. The model of the Sensor Mission is depicted in Figure 9.

The core of the Sensor Mission is a set of *SimpleProcess* elements which represent the different operations that will be performed by the observation platform’s acquisition software. The operations may include data retrieval through sensor commands, post-measurement operations and data storage. Using the *typeOf* property, these *SimpleProcess* can be identified as instances of a specific sensor command (defined in the Sensor Instance) or a built-in SWE Bridge function (defined in the SWE Bridge Model). To allow a flexible configuration, an array of settings may be included, which may modify the default values inherited from the parent process.

The instantiated processes can be connected among them using *connections*, creating chains of processes. These chains of processes contain all operations required by the acquisition process: data retrieval, data processing and data storage. This provides the user a highly flexible framework to configure an acquisition process based on standard SensorML files.

### 4.4. Sensor Deployment Files and SOS Registration

SDF are mainly focused to address the operational interoperability challenges detected when integrating a new sensor to an observation platform (requirements 2–4, Section 2.1). However, when registering a new sensor resource to a Sensor Observation Service, a different procedure is needed. Although an SDF contains all the required metadata to register a sensor to an SOS server, it needs to be mapped to the transactional SOS operations.

**Figure 10:**
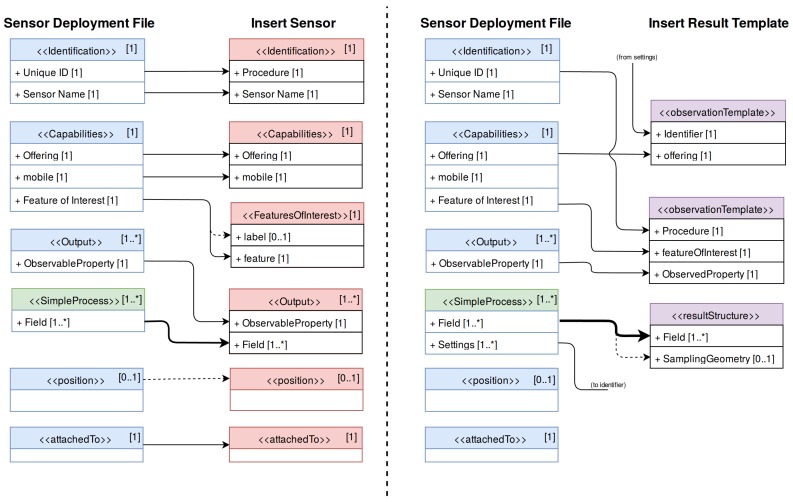
Metadata mapping from Sensor Deployment Files (SDF) to the Sensor Observation Service (SOS) transactional operations *InsertSensor* (**left**) and *InsertResultTemplate* (**right**). The blue elements correspond to Sensor Instance elements while the green elements correspond to Sensor Mission elements. Dashed lines indicate optional elements and thick lines indicate multiple elements.

These operations are *InsertSensor*, which registers the sensor metadata, and *InsertResultTemplate*, which registers the data structure and the encoding of the sensor’s observations. If the sensor was previously registered in another deployment in the same SOS server it is possible to modify the sensor metadata by using the *UpdateSensorDescription* operation. The information contained within a SDF has to be mapped to those SOS operations, as shown in Figure 10. After this workflow, a sensor has been registered to an SOS server, so that the upload of the measured data can be started through the *InsertResult* operation.

## 5. Standards-Based Universal Acquisition Middleware

### 5.1. Background

In the previous section an approach how to organize sensor metadata into standardized SensorML-encoded SDF has been discussed. However, in order to provide a plug and play framework, a middleware capable of automatically retrieving this SDF, interpret it and configure an acquisition the process is required.

Some marine observation platforms are deployed in long-term missions in remote and inaccessible places (i.e., underwater gliders and profilers). Therefore, these platforms present severe power and communications constrains. Taking into account these constraints and aiming to achieve a highly interoperable and versatile software component, the following design requirements where formulated for such a middleware:

- **Plug and play sensor discovery**: The middleware shall be able discover and communicate with sensors connected on-the-fly, without any prior knowledge about these sensors.
- **Standards-based configuration**: The middleware shall be able to interpret SDFs and setup an acquisition process based on the information contained in these files.
- **Cross-platform design**: The middleware shall be deployable in a maximum number of platforms, regardless of their particular hardware and software architecture.
- **Minimum resource requirements**: Due to the intrinsic constraints of some observation platforms, the usage of hardware and software resources has to be reduced as much as possible (RAM usage, bandwidth, etc.).
- **Standard compliance**: Such a middleware shall be described through SensorML files to allow systems to automatically understand its role and capabilities.

The Sensor Web Enablement Bridge (SWE Bridge) is a middleware component designed to fulfil these previously mentioned requirements. This middleware is aimed to be used as a universal driver for any sensor providing a RS232 or Ethernet interface. Its cross-platform and hardware abstraction design makes it suitable to be deployed in the majority of observation platforms, whether they are fixed or mobile.

### 5.2. SWE Bridge Model

**Figure 11:**
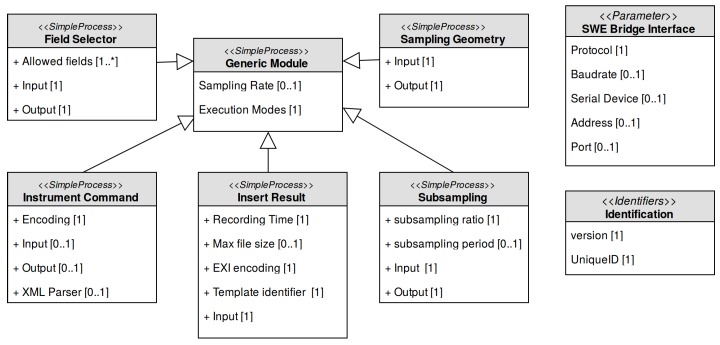
SWE Bridge model. All modules inherit from the generic module, where the execution options for the processes are defined. Then each module expands its definition with its particular settings. The model also contains a set of identifiers and a generic communication’s interface.

The SWE Bridge has been designed following a SensorML-like style, implementing computational equivalents for the SensorML elements (*SimpleProcess*, *Parameters*, *DataRecord*, etc.) and also providing SensorML-based inheritance and configuration mechanisms (*typeOf* and *Settings*). Due to this approach, the SWE Bridge can also be modelled using the SensorML standard. The ultimate goal of this model, named SWE Bridge Model, is to provide an unambiguous description of this middleware, understandable by automated processes, providing a framework to automatically generate SDFs with minimum human intervention. This model is shown in Figure 11 and it is available online at [39].

The SWE Bridge model defines a generic communication interface that abstracts the physical layer, providing a protocol-agnostic communication functionality to the rest of the software. The supported protocols are serial communication, TCP and UDP.

The SWE Bridge’s core is its set of modules, which are templates for observation-related, built-in processes. These processes include data retrieval, simple data manipulation and data storage among others. All these operation are described with *SimpleProcess* elements, which can be instantiated, configured and connected within a Sensor Mission as detailed in Section 4.3. Each module can be instantiated to create a process that will be executed on runtime.

The generic module is an abstract module that contains the necessary information for the coordination and operation of the resulting processes. This information is encapsulated in a set of flags named Execution Modes, whose main function is to control the circumstances under which a particular process shall be executed.

Derived from this generic module the following built-in modules are implemented in the current version of the SWE Bridge:

- **Instrument Command**: This module provides a unified process to communicate with a sensor. Depending on the configuration, this module can be used to send any kind of commands and/or receive sensor data.
- **Field Selector**: This module allows to filter the response of a sensor, selecting the desired information and discarding the rest.
- **Subsampling**: This module allows to create subsampled data sets. It is especially useful in platforms with severe communication constraints, where a subsampled data set is transmitted in real time and a full data set is stored locally.
- **Sampling Geometry**: This module adds the platform position to a data structure, correlating sensor data with the platform’s coordinates.
- **Insert Result**: This module stores the incoming data to standard O&M files, encoded in XML or EXI.

Depending on the module, it may have an input, an output or both. If a module does not have an input, it represents the beginning of the process chain (i.e., Instrument Command). On the contrary, if it has only an input and does not have output this process represents the end of a process chain (i.e., Insert Result). If a module has both input and output, the module is an intermediate process, performing data manipulation/processing. A *DataRecord* structure emulating the SWE Common Data Model standard is also used to pass data from process to process [38]. It is also possible to expand the SWE Bridge functionalities by implementing custom modules. A new module shall also inherit the parameters from the SWE Bridge generic module and follow the same data structure and input/output logic. In Section 6 different process chains for different real-world use cases are presented, including custom modules.

### 5.3. Implementation

**Figure 12:**
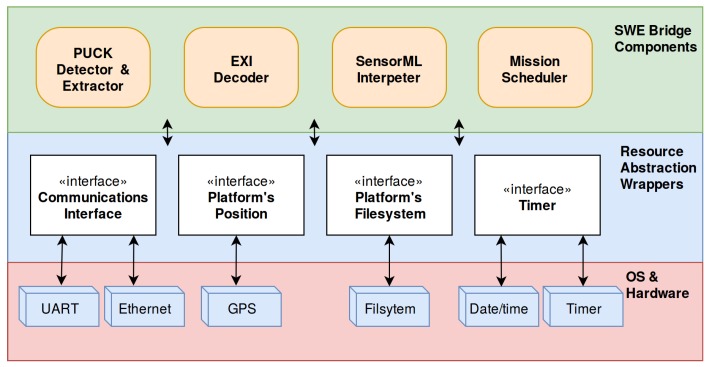
SWE Bridge internal architecture. The different components use the resource abstraction wrappers to hide the underlying hardware and operating system, providing a unified way of accessing platform-dependent resources.

In order to fulfil the cross-platform and minimum resources requirements, the SWE Bridge has been implemented using ANSI C, with special emphasis on minimizing the usage of underlying software and hardware resources. Its implementation is available online at [40]. All platform-dependent resources are abstracted using resource abstraction wrappers, which provide an unified way to access the platform’s resources (see Figure 12). These wrappers are the only functions that need to be adapted when deploying the SWE Bridge in a new platform.

The SWE Bridge operation is organized in four components that are executed sequentially: the OGC PUCK Detector & Extractor, the EXI decoder, the SensorML Interpreter and the Mission Scheduler. The hardware resources needed by these components are: access to a serial and/or Ethernet communication interface, access to the observation platform’s coordinates (if the platform is mobile), access to the platform’s filesystem and a timer to schedule internal operations.

**Figure 13:**
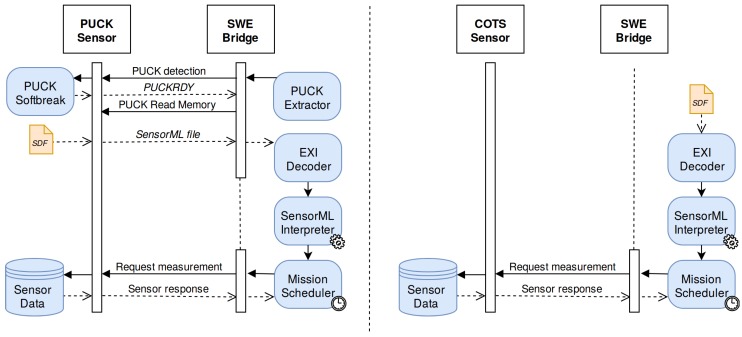
SWE Bridge operation. On the left the operation with a OGC PUCK-enabled sensor is shown. On the right the operation of a COTS sensor is presented, with the SDF stored locally on the platform.

The execution of the SWE Bridge may vary slightly depending on whether the sensor is OGC PUCK-enabled or not, as shown in Figure 13. OGC PUCK-enabled sensors shall provide their SDF embedded within their payload memory. However, when interfacing a COTS sensor its associated SDF shall be uploaded to the platform filesystem.

The OGC PUCK Detector and Extractor detects new OGC PUCK-enabled sensors connected to the communications interface. Once a new sensor is detected, this component extracts its SDF. When interfacing a COTS sensor, a local SDF can be passed as argument to the middleware, bypassing the OGC PUCK Detector and Extractor component.

The EXI decoder, based on the EXIP framework, extracts and stores the desired information from the SDF into an intermediate structure [41]. Using a set of rules, the decoder identifies potentially useful elements to the SensorML Interpreter Service. The SensorML Interpreter service takes the extracted information and uses this data to configure the acquisition process. The first step is to configure a communication interface according to the information decoded sensor description. Afterwards this service examines the set of *SimpleProcesses* that are defined within the Sensor Mission and generates a process instance for each one of them. The generated processes are connected according to the SDF’s *connections* section and finally the internal parameters of these processes are configured as specified in the *Settings* section.

Once the Auto-configuration Service has setup all necessary processes in the SWE Bridge, the Mission Scheduler is started. This is a timer-based scheduler that manages and controls the execution of the previously configured process chains.

## 6. Use Cases

This section illustrates different use cases where the combination of SDFs with the SWE Bridge middleware are used to successfully enable and demonstrate the plug and play integration of sensor into Sensor Web Enabled architectures.

### 6.1. NeXOS Project

The NeXOS project was an EU-funded project that aimed to develop cost-effective, innovative and compact multifunctional systems which can be deployed on fixed and mobile platforms [42], with special emphasis on interoperability and SWE-based architectures. Within this project the acquisition chain presented in Section 3 was arranged into the Smart Electronic Interface for Sensor Interoperability (SEISI) [43]. Different demonstration mission where performed using different sensors deployed on platforms such as gliders, underwater observatories, buoys and profilers among others. In this section the focus is put on the integration of two different NeXOS sensor developments into the SeaExplorer Glider.

**Figure 14:**
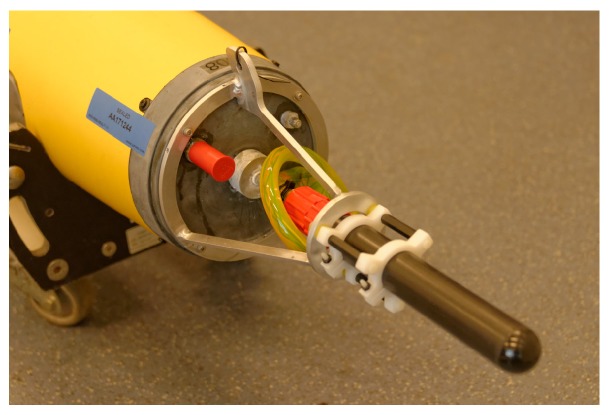
NeXOS A1 Hydrophone integrated on the SeaExplorer glider as payload.

Two different NeXOS-developed sensors where deployed in the SeaExplorer Glider [44], the Mini.1 and the A1 Hydrophone. The Mini.1 is an optical sensor that measures hydrocarbon concentrations in water while the A1 Hydrophone is a smart acoustical sensor with embedded real-time processing capabilities for noise measurements and mammal detection (shown in Figure 14). Both sensors implemented the OGC PUCK protocol and had their own SDF embedded within their respective payloads, describing the sensors and their mission. These files are available at [37].

**Figure 15:**
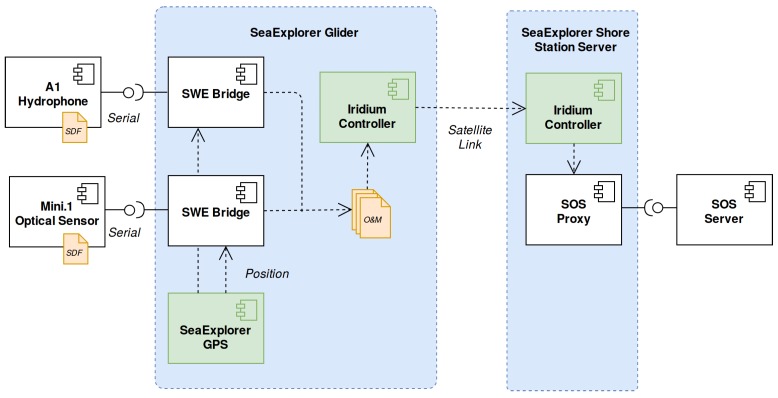
SeaExplorer mission acquisition chain

Within the SeaExplorer controller, which runs an embedded Linux operating system, two instances of the SWE Bridge software were executed. These instances where in charge of retrieving and interpret the SDF and setup the data acquisition process. As the SeaExplorer glider is a mobile platform, a resource abstraction wrapper was developed in order to relate the acquired data with the vehicle position, based on socket communication between the GPS driver and the SWE Bridge. The SeaExplorer used an Iridium satellite link to communicate with the shore station. The management of this power-consuming and low-bandwidth communications in power-constrained platforms is critical. Thus, the satellite-link is controlled by the platform operators, deciding when and how the generated files will be sent to shore. The acquisition chain is showed in Figure 15.

**Figure 16:**
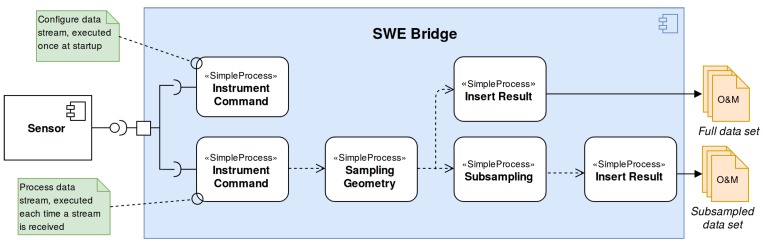
SWE Bridge mission scheduler configuration for the SeaExplorer NeXOS mission.

**Figure 17:**
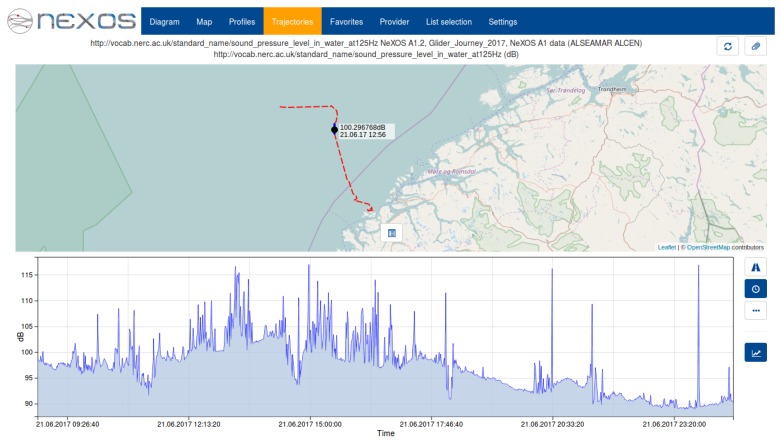
Sound Pressure Level (SPL) at 125 Hz octave band acquired by a SeaExplorer glider in a mission at the Norwegian coast. The incoming data from the hydrophone was subsampled and sent to shore in near real-time during deployment.

The SWE Bridge generated two sets of data files: a subsampled data set sent in near real-time through the glider’s satellite-link and a full data set, stored locally and recovered with the glider at the end of the mission. In Figure 16 the SWE Bridge mission scheduler configuration workflow is shown. The data is retrieved from the sensor by the Instrument Command process. Afterwards the Sampling Geometry process associates the latest platform position to each measurement. The data is then passed to two different branches. The first branch sends the data to an Insert Result process, which stores the full data set locally. The second branch subsamples the incoming data before passing it to another to another Insert Result process. This process stores the subsampled data set into O&M files, which is transmitted to a shore station through the glider’s satellite link. A subsampled acoustic noise data set gathered by the A1 Hydrophone sent in near-real during field trials can be seen at Figure 17.

### 6.2. EMSODEV Project

**Figure 18:**
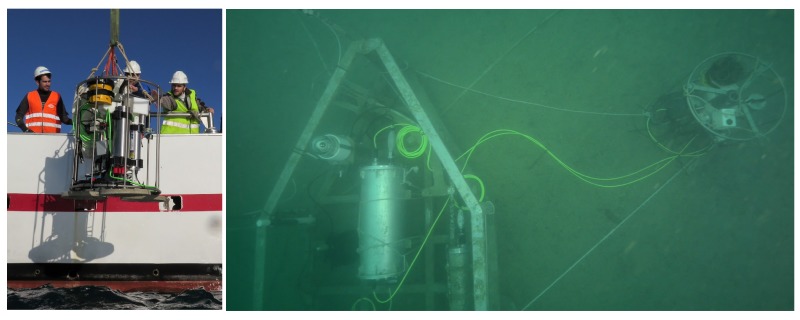
EMSO Generic Instrument Module (EGIM) with its instrument pack during its deployment (**left**) and EGIM deployed at the OBSEA observatory (**right**).

The proposed acquisition chain has also been used within the EU-funded project EMSODEV. This project aims to develop the EMSO Generic Instrument Module (EGIM), as well as its associated data infrastructure. The EGIM is a compact-sized observation platform designed for long-term deployments at the EMSO nodes [45]. Its main purpose is to gather extensive, multidisciplinary data sets in a standardized fashion. In order to archive and distribute the acquired data, an SWE-based infrastructure is implemented. For collecting this multidisciplinary data, the EGIM includes an instrument pack of COTS sensors, shown in Table 1. The EGIM device with its instrument pack is shown in Figure 18.

The EGIM has two modes of operation: autonomous and cabled. While in autonomous mode, the EGIM is powered by internal batteries and the data coming from the instruments is logged into CSV files, retrieved after the device recovery. On the contrary, when it is operated in cabled mode, it draws power from an external source, communicating the data in real-time using an Ethernet link. The EGIM implements a serial to Ethernet converters for each sensor, which are operated externally by an acquisition server.

**Figure 19:**
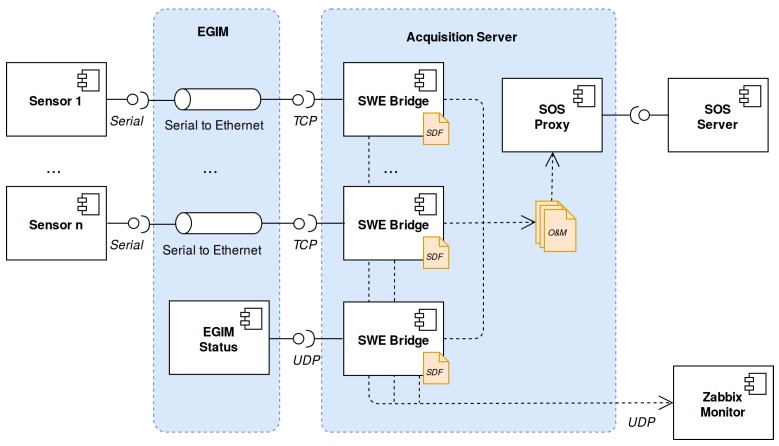
EGIM cyber-infrastructure in a cabled mode scenario.

The EGIM device itself also sends data regarding its internal status using UDP frames. These frames contain information about input voltage, input current, remaining storage capacity, internal temperature and a leak detection alarm. Within the EMSODEV project, interoperability was also a matter of great concern. Thus, an SWE-based data acquisition process with the components presented in Section 3 was implemented [46]. Figure 19 shows the EMSODEV cyber-infrastructure in a cabled mode scenario. Each sensor is attached to the EGIM using RS232 ports, which converts this serial communication to an Ethernet link, providing a TCP/IP interface.

The acquisition server runs an instance of the SWE Bridge for each instrument deployed on the EGIM node (except the hydrophone, which records data in its internal memory). A SDF file has been written for each sensor, available at [37]. As the sensors do not implement the OGC PUCK protocol (COTS sensors), these SDFs are stored locally in the acquisition server. Another instance of the SWE Bridge is also used to decode the EGIM internal status frames, which are treated as scientific data. The SWE Bridge generates O&M files containing the acquired data. These files are passed to an SOS proxy, which injects the data to an SOS server.

Within the EMSODEV project, the use of a Zabbix monitoring system to monitor the status of the EGIM and its associated cyber-infrastructure was proposed [47]. This monitoring system does not support O&M-based transactions. Therefore, in order to fulfil the architecture requirements, the SWE Bridge functionality was expanded by implementing a specific module to send instrument data to a Zabbix monitoring server using its specific format. This module, called Send to Zabbix, sends an UDP frame to a Zabbix server for each new value arriving from the sensors.

**Figure 20:**
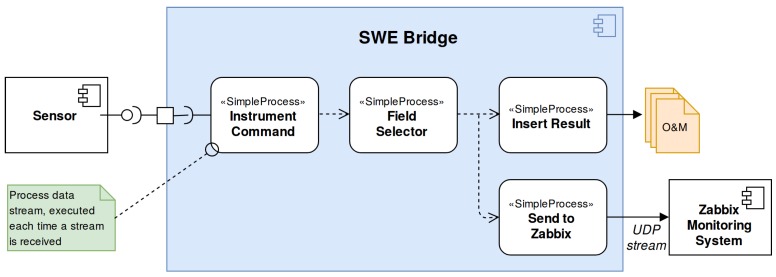
Mission scheduler configuration used by the SWE Bridge instances deployed at the EMSODEV acquisition server. The data coming from each sensor (all of them configured in stream mode) is acquired using an Instrument Command process, which then passes the data to a Field Selector process. This process filters the useful data and discards the undesired variables, depending on the communications protocol of each sensor. Later on, two different branches are created, one that stores the data into Observations and Measurements (O&M) files using the Insert Result process, and another one that sends the data to the Zabbix Monitoring System.

Except the hydrophone, all the sensors including the EGIM itself are configured in streaming mode. Thus, each SDF use the same process chain for the SWE Bridge’s mission scheduler, shown in Figure 20.

A first field test of the EGIM developments at the OBSEA underwater observatory was conducted from 1 December 2016 to 15 April 2017 [48]. The test showed that the cyber-infrastructure was robust and interoperable as new sensors can be easily deployed, just plugging a new sensor to an EGIM’s empty slot and writing a new SDF.

### 6.3. INTMARSIS Project

The INTMARSIS project aims to monitor underwater seismic activity in real-time, allowing a precise estimation of actual earthquake scales. To achieve this goal a stand-alone seismic system with real-time telemetry was designed and tested [49]. One further objective was to assess the usability of OGC compliant standardized acquisition chains in underwater seismic applications.

**Figure 21:**
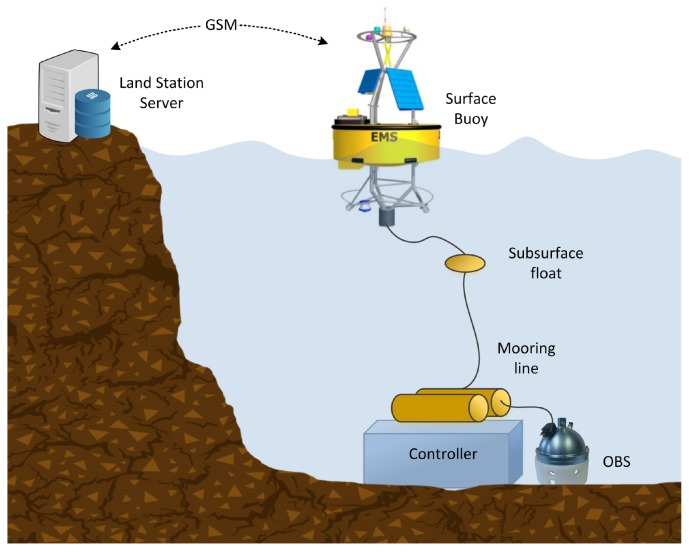
INTMARSIS System overview. At the seafloor the OBS (Ocean Bottom Seismometer) acquires seismic data, storing it locally. A subsampled set of data is sent through the mooring line using an inductive modem. The surface buoy receives the real-time subsampled seismic data, which is transmitted to the Land Station server using a GSM link

**Figure 22:**
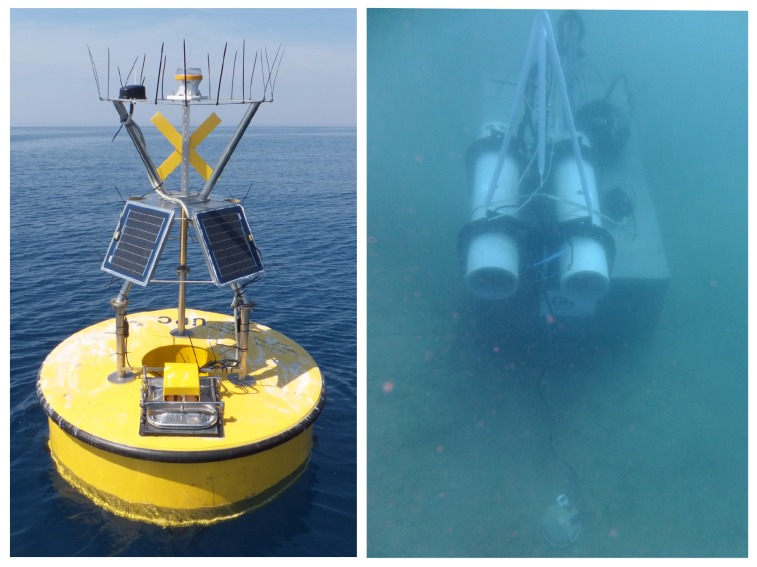
INTMARSIS buoy (**left**) and INTMARSIS Ocean Bottom Seismometer (**right**).

The INTMARSIS system, shown in Figure 21, is composed of mainly two components, an ocean bottom seismometer (OBS) and a surface buoy. The communication between the OBS and the surface buoy is performed by a stainless steel mooring line using inductive modems (SeaBird Electronics UIMM). This inductive modem provides low-bandwidth half-duplex communication through mooring lines (up to 7000 m) where regular cables are not practical [50]. The INTMARSIS system, deployed near the Catalan coast, is shown in Figure 22.

The OBS acquires 3 channels (X, Y and Z axis), taking 125 Samples per second (SPS) with a precision of 24 bits. However, due to the low bandwidth provided by the inductive modems (1200 bps), the full data set is not transmitted in real-time, but stored locally. The OBS controller generates a subsampled data set at 25 SPS which is transmitted thorough the inductive modem.

**Figure 23:**
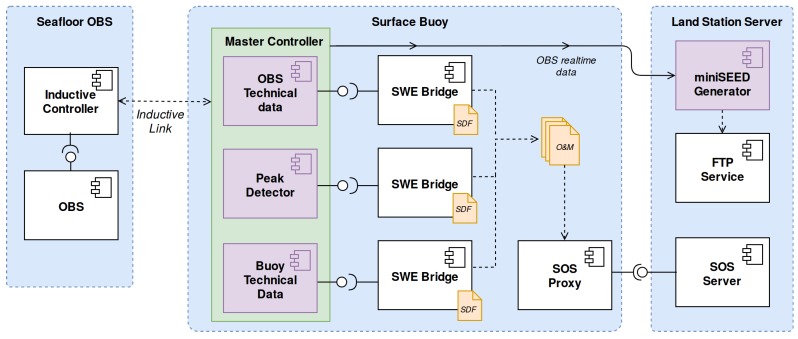
INTMARSIS System acquisition chain. Virtual instruments are depicted as purple components.

In Figure 23 the INTMARSIS acquisition chain is depicted. The subsampled data set, alongside with OBS’s technical data, is sent to the buoy through the inductive link. The inductive communication and the processing of the acquired data as well as internal sensors is performed by the master controller, hosted by the surface buoy. This software component is modelled as three different Virtual Instruments (VIs): OBS technical data, buoy technical data and a peak detector.

Technical data from both OBS and buoy include internal temperature and humidity gathered by low-cost sensors integrated at the electronics board. Although this data is not scientifically relevant it may prove useful to the operators to detect hardware malfunctions and water leaks. The master controller collects the internal data and aggregates it into two different UDP streams, one for the buoy technical data and another one for the OBS technical data.

The peak detector processes the seismic data and returns the maximum absolute value during a period of time (10 s by default). Each of these VIs is interfaced by an instance of the SWE Bridge and its data sent to an SOS server at the shore station using a GSM modem. The SDF associated with each VI are available at [37].

The raw seismic data is sent in near real-time to the land station, where it is processed and stored in miniSEED files (a standard format for seismic data). This data is made publicly available through a FTP server. Although the raw seismic data is not stored by the SOS server, the peak values time series provides an indicator of the seismic activity. As the volume of data is several orders of magnitude lower than the raw seismic data, it is much easier to archive and display this data in Sensor Web environments. With this approach it is possible to discover and access data from the seismometer using Sensor Web components, and only download the seismic events instead of the whole data set.

### 6.4. SWE Bridge Performance

As discussed in Section 2.1, many observation platforms present severe constraints in terms of power supply and computational resources. Although the SWE Bridge software is not performing any computationally expensive operations, it makes extensive use of dynamic memory due to the arbitrariness of SensorML documents. In this section the assessment of the SWE Bridge performance is presented when interfacing the sensors deployed in NeXOS, EMSODEV and INTMARSIS projects. In order to obtain comparable results, all tests were performed in a Raspberry Pi single board computer (specifications shown in Table 2), acting as a host controller for the SWE Bridge. The performance was assessed using the Massif heap memory profiler [51].

**Figure 24:**
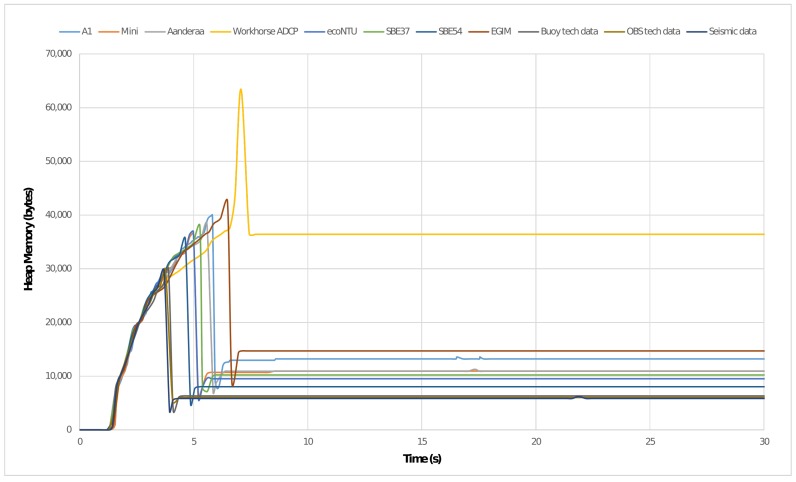
SWE Bridge time-based memory profile when interfacing sensors from NeXOS, EMSODEV and INTMARSIS projects. The peak usage of heap memory is registered when decoding the SDF file. Afterwards, during the operation stage, the memory consumption is kept constant.

The execution of the SWE Bridge can be classified in two differentiated stages: setup (which includes the components *OGC PUCK Extractor*, *EXI Decoder* and *SDF Interpreter*) and operation (*Mission Scheduler*). During the setup process the SDF is decoded and interpreted, which produces a peak in the use of dynamic memory. Once the setup is finished, a significant amount of memory is freed and the use of dynamic memory is maintained low and constant during the operation stage. This behaviour can be observed Figure 24, where the first 30 seconds of a time-based memory profile is depicted.

The amount of memory used at the stationary stage is mainly dependant on the nature of the sensor response and the mission complexity. Large responses require larger buffers and each process within the SWE Bridge also increases the usage of dynamic memory. The peak usage of dynamic memory as well as the average values in stationary phase are shown in Table 3.

The overall computational load, measured in kilo instructions per second (KIPS), is mainly dependant on the sensor’s communications protocol, data stream period and response length. Sensors using TCP/UDP protocols increment significantly the computational load when compared to serial sensors. Moreover, sensors with short periods of data stream with large responses present the higher usage of CPU (i.e., SBE54 and Aanderaa 4831).

## 7. Conclusions and Future Work

In this work a framework to enable plug and play sensor integration into research data infrastructures have been proposed. It is based on the combination of different standards from the OGC’s SWE framework and the W3C consortium. Using these standards a set of interoperable components have been presented to bridge between any kind of (in-situ) sensor and the Sensor Web. To ensure re-usability of the results aspects such as cross-platform support and the use of open source licenses were emphasized. These components can be easily adapted to different scenarios without any significant modification, overcoming the intrinsic constraints of ocean observation platforms.

Sensor’s metadata, as well as deployment and acquisition-specific metadata are combined in a coherent format by using the concepts of SDF, providing a SensorML-based template for unambiguous sensor deployment and sensor operation description. The advantages of the combination of SDFs with the proposed acquisition chain (SWE Bridge, SOS Proxy and SOS server) has been demonstrated in three real-world scenarios. These deployments include a mobile platform with severe power and communications constraints (SeaExplorer Glider), a multidisciplinary fixed-point observation platform with a pack of commercial sensors (EGIM), and a complex seismic system (INTMARSIS system). Different sensors where integrated into observation platforms, including six COTS sensors, two newly developed OGC PUCK-enabled sensors and a complex seismic system (treated as three Virtual Instruments), each one of them with their own non-standardized proprietary protocols.

The design of the presented components and their SensorML descriptions provide the foundations for generating automated processes that can combine sensor metadata and acquisition chain metadata so that SDFs can even be created in a semi-automated manner.

Further work in this field should be focused on facilitating the application of the presented sensor acquisition chain by making the generation of SDFs easier. For example developing a user-friendly graphical user interface to generate SDFs with minimal human intervention. This tool should be able to combine the information from the sensor, the description of the acquisition chain’s components and the capabilities of SOS servers in order to allow users to generate their own configuration files in an intuitive manner, hiding the specificities of the SWE standards from the end user. This would allow users to leverage the potential of the Sensor Web without the need of in-depth knowledge of complex standards and services required.

## Figures and Tables

**Table 1:** EGIM Instrument Pack. The communications refers to the communications interface that the server uses to communicate to the sensors.

Sensor Type	Sensor Name	Manufacturer	Communication Link
CTD	SBE 37	SeaBird Electronics	TCP/IP
Tsunami Meter	SBE 54	SeaBird Electronics	TCP/IP
Oxygen Optode	Aanderaa 4831	Aanderaa	TCP/IP
Turbidimeter	Eco NTU	Wetlabs	TCP/IP
ADCP	Workhorse	Teledyne	TCP/IP
Hydrophone	icListen	Ocean Sonics	FTP
EGIM Internal Stuatus	EGIM	EMSODEV Consortium	UDP

**Table 2:** Raspberry Pi 2 specifications.

Processor Architecture	ARM 7
Processor Speed	900 MHz
Number of Cores	4
RAM memory	1 GB
Operating System	Raspbian Jessie Lite (version July 2017)

**Table 3:** SWE Bridge performance assessed when interfacing sensors from the NeXOS, EMSODEV and INTMARSIS projects. The average values of the memory consumption are calculated in the stationary phase (after the setup).

Sensor Name	Sensor Parameters				SWE Bridge Performance			
	Protocol	Stream (bytes)	Period (s)	SDF Size (bytes)	Max Heap (kBytes)	Avg Heap (kBytes)	Avg Stack (kBytes)	CPU Load (KIPS)
A1	Serial	61	1	3158	40.05	13.26	1.631	35.23
Mini.1	Serial	68	1	2451	36.63	10.96	1.568	34.50
Aanderaa 4831	TCP	80	1	3440	38.36	10.93	1.029	88.36
Workhorse	TCP	688	60	9431	63.66	36.43	0.830	78.23
Eco NTU	TCP	32	1	3290	36.93	9.53	0.892	78.26
SBE 37	TCP	72	10	3429	37.91	10.22	0.740	68.39
SBE 54	TCP	140	1	2769	35.56	8.05	0.895	87.35
EGIM	UDP	137	20	4477	42.57	14.70	0.691	68.03
Seismic Data	UDP	10	10	1728	29.96	5.84	0.692	63.61
OBS Technical	UDP	30	30	1764	29.76	6.02	0.683	63.75
Buoy Status	UDP	30	30	1764	29.96	6.32	0.685	63.89

## Abbreviations

The following abbreviations are used in this manuscript:

OGC	Open Geospatial Consortium
SWE	Sensor Web Enablement
O&M	Observations and Measurements
SOS	Sensor Observation Service
SensorML	Sensor Model Language
EXI	Efficient XML Interchange
SDF	Sensor Deployment File
COTS	Commercial off-the-shelf
